# Phenotypic mismatch between suspects and fillers but not phenotypic bias increases eyewitness identifications of Black suspects

**DOI:** 10.3389/fpsyg.2024.1233782

**Published:** 2024-04-12

**Authors:** Jennifer M. Jones, Jacqueline Katzman, Margaret Bull Kovera

**Affiliations:** ^1^Department of Psychology, The Graduate Center, City University of New York, New York, NY, United States; ^2^Department of Psychology, John Jay College of Criminal Justice, City University of New York, New York, NY, United States

**Keywords:** phenotypic bias, own-race bias, cross-race, eyewitness identification, lineup construction, suspect bias, lineup fairness

## Abstract

**Introduction:**

Despite converging evidence that people more closely associate the construct of criminality with Black people who exhibit a more African facial phenotype than Black people who express a more European phenotype, eyewitness researchers have largely ignored phenotypic bias as a potential contributor to the racial disparities in the criminal legal system. If this form of phenotypic bias extends to eyewitness identification tasks, eyewitnesses may be more likely to identify Black suspects with an African rather than European phenotype, regardless of their guilt status. Further, in cases where the witness’s description of the perpetrator does not contain phenotypic information, phenotypic mismatch between the suspect and the other lineup members may bias identification decisions toward or against the suspect. If witnesses can use elements of the lineup construction to guide their identification decisions rather than relying on their recognition memory, then the lineup should be deemed unfair due to suspect bias. The current study also investigated lineup presentation method as a procedural safeguard, predicting that that when lineups were presented simultaneously, there would be a significant two-way interaction of phenotypic bias and lineup composition, with a larger simple main effect of phenotypic bias when lineups were suspect-biased (i.e., the fillers were a phenotypic mismatch to the suspect) than when all lineup members shared the same phenotype. We expected that this interaction would be significantly smaller or non-significant for sequential lineups.

**Methods:**

Participants watched a mock crime video that contained a Black culprit with either a more African phenotype or a less African phenotype before attempting identifications from a photo array that contained a suspect whose phenotype always matched the culprit viewed in the video, but varied in culprit-presence, phenotypic match of the suspect and fillers, and presentation method.

**Results:**

Participants did not identify Black suspects with Afrocentric features more often than Black suspects with Eurocentric features. However, witnesses made more identifications of suspects when the fillers did not match the suspect’s phenotype compared to when all lineup members possessed similar phenotypic features.

**Discussion:**

In sum, phenotypic bias did not influence our participant-witnesses’ identification decisions, nor interact with lineup composition and lineup presentation type to affect identifications of suspects, suggesting that phenotypic bias may be less influential in match-to-memory tasks than other types of legal decision-making (e.g., determining guilt and sentencing). However, the suggestiveness created by failing to match fillers’ phenotypes to the suspect’s phenotype can be avoided with proper attention to fair lineup construction.

## 1 Introduction

Mistaken eyewitness identification has been identified as the leading contributing factor to wrongful convictions, as evidenced by 69% of DNA exoneration cases involving a mistaken identification (Innocence Project, 2023). Moreover, there are great racial disparities in these data as 65% of misidentified exonerees were Black defendants (The National Registry of Exonerations, 2022). Scholars who have explored the contribution of race to mistaken identifications have almost exclusively examined the role of an own-race bias in identification accuracy (Katzman and Kovera, 2023). Also known as the cross-race effect, people are more accurate when identifying people from their own racial group than they are identifying people of other racial groups (Meissner and Brigham, 2001a; Lee and Penrod, 2022; Katzman and Kovera, 2023). Yet the own-race bias does not provide a sufficient explanation for these disparities in wrongful convictions because (a) five times as much crime occurs with victims and perpetrators of the same race versus different races (National Archive of Criminal Justice Data, 2016), (b) the size of the own-race bias in identification accuracy is small relative to the large racial disparities in the exoneration data, and (c) a meta-analysis of the own-race bias literature demonstrated that both White and Black participants were better able to discriminate among previously seen White than Black faces (Katzman and Kovera, 2023). Thus, to reduce wrongful convictions, it is important to explore additional psychological mechanisms that may explain how race contributes to eyewitness identification accuracy.

### 1.1 Beyond own-race bias: external influences on witnesses’ mistaken identifications of Black faces

Moreover, it is not a new proposition that memories for perpetrators might be altered through external events like the process of providing a description of a face (Schooler and Engstler-Schooler, 1990; Meissner and Brigham, 2001b; Alogna et al., 2014), exposure to mugshots (Deffenbacher et al., 2006), or the act of engaging in repeated identification procedures (Wells et al., 2020; Wixted et al., 2021). Witnesses’ decision-making processes are susceptible to influence from external cues, particularly when they do not experience an immediate sense of recognition (Bradfield et al., 2002). For example, both the selection of dissimilar fillers and differences between photos of fillers and the suspect (e.g., background and clothing) in a photographic lineup cause witnesses to be more likely to identify the suspect, irrespective of the suspect’s guilt (for a review, see Wells et al., 1998; Wells et al., 2020 for a review). Rather than distorting or degrading witnesses’ memory for the perpetrator, these factors affect decision-making through creating suspect bias in the lineup procedure (Smalarz, 2021). If a lineup procedure biases a witness toward identifying the suspect through non-memorial cues, the identification procedure could be deemed unfair and inadmissible in court, as the legal system requires that an eyewitness identification must be based on the independent memory of the witness, free from suggestive influences (*Perry v. New Hampshire, 2012*). A suspect-biased lineup not only puts innocent suspects at greater risk of being misidentified, but also fails to provide guilty culprits with due process. Eyewitness researchers have made a distinction between two types of factually correct identifications: *legitimate hits*, where the witness makes a correct identification based on their memory of the culprit, and *illegitimate hits*, where suggestive procedures ultimately produce a correct identification (Wells et al., 2012).

Internal cognitive structures of witnesses, like racial stereotypes, may also alter witnesses’ identification decisions. One potential internal phenomenon that may explain racial disparities in misidentifications that has received relatively little attention among eyewitness researchers is phenotypic bias. *Phenotypic bias* refers to stereotypes, prejudice, and discrimination based on race-related facial characteristics. Whereas racial bias involves comparisons between different racial groups (e.g., White people vs. Black people), phenotypic bias involves comparisons between people of the same racial group who possess varying phenotypic characteristics (e.g., light-skinned Black people vs. dark-skinned Black people). This form of subgroup prejudice has manifested in several phenomena, including *colorism* (Okazawa-Rey et al., 1987; Russel et al., 1992), *Afrocentric bias* (Blair et al., 2002), and *bleaching syndrome* (Hall, 1994, 1995). Previous research investigating the effect of skin tone bias on perceptions of Black people in the United States has revealed that individuals who possess features that are more typical of their racial group are perceived and treated more negatively (Maddox, 2004).

Phenotypic bias is rooted in the fact that criminality is a central feature of the stereotype that people have about Black individuals, irrespective of whether people are highly prejudiced toward that group (Devine, 1989) or whether Black people are members of their in-group (i.e., other Black people) or out-group (e.g., White people; Maddox and Gray, 2002). Black stereotypes are automatically activated with exposure to Black people, but may be more strongly activated by some members of that group than others as there is variation in the extent to which Black people exhibit a facial phenotype that is associated with being African. Black people who have more phenotypically African facial features (e.g., darker skin, fuller lips, wider nose, afro-textured hair, and prominent brow) are viewed as more representative of their race (Knuycky et al., 2013) and thus activate racial stereotypes more strongly than do those whose features are more phenotypically European (Blair et al., 2002, 2004b; Maddox, 2004). People with more Afrocentric facial features are more likely to prime stereotypes about Black aggressiveness (Blair et al., 2005) and criminality (Eberhardt et al., 2004; Knuycky et al., 2013), and be seen as more intimidating than people with less Afrocentric features (Kleider-Offutt et al., 2018). It is difficult to consciously inhibit criminal inferences activated by Afrocentric features, even when aware of the consequences associated with phenotypic bias (Blair et al., 2004a).

Phenotypic bias has detrimental consequences for the treatment of Black people with Afrocentric features within the criminal justice system. For example, laypeople and police officers infer that Black people with Afrocentric features are more likely to engage in criminal activities (Eberhardt et al., 2004; Kahn and Davies, 2011). In studies employing shooting simulations to study shooter bias—the tendency to mistakenly shoot unarmed Black men and fail to shoot armed White men—both Black and White participants were more likely to show shooter bias when the Black men in the stimulus materials had more Afrocentric features compared to Eurocentric features (Kahn and Davies, 2011). Among Black people found guilty of a crime, those with more Afrocentric features receive harsher punishments, including the death penalty, than those with less Afrocentric features (Blair et al., 2004b; Eberhardt et al., 2006; Peterson, 2016). Moreover, in an archival study of use of force cases, independent raters coded the phenotypic stereotypicality of each suspect’s booking photograph and found that the more phenotypically White an individual was perceived to be, the less police force was used during the interaction. In other words, police used less force with highly stereotypical White people compared to less stereotypically White people, resulting in a pro-White protective bias (Kahn et al., 2016). Taken together, there is converging evidence that people make inferences about others’ culpability and deservingness of punishment based on their phenotypic features.

Less is known about whether this form of phenotypic bias extends to the recognition memory and decision-making processes involved in eyewitness identification. In one study, participants viewed a series of slides depicting a Black man leaving a building and were told that he was accused of committing either a stereotypically White or stereotypically Black crime (Osborne and Davies, 2013). Participants were then asked to identify the man from a series of 100 pictures that were created by morphing the target face with a face that was more phenotypically African and with a face that was less phenotypically African than the target face. The 50th picture in the series was the target face. Participants who were told that the target was committing a stereotypically Black crime chose a morphed picture that was more phenotypically African and significantly different from the target face.

In another series of experiments evaluating misidentifications in lineup identification procedures, witnesses perceived Black faces with more African phenotypes as more familiar than Black faces with a less African phenotype (Knuycky et al., 2013, Experiment 2), and made more identifications from culprit-absent lineups when they contained more, rather than fewer, lineup members with phenotypically African features (Knuycky et al., 2013, Experiment 3). This finding could be explained by the tendency for people to mistakenly report novel faces as previously seen more often when the novel faces are “typical” rather than “distinctive” (Vokey and Read, 1992; Dewhurst et al., 2005), combined with the finding that witnesses perceived Black faces with more African phenotypes as more “prototypical” of Black faces (Knuycky et al., 2013, Experiment 1). Although these studies are provocative, none of them were conducted within an eyewitness identification paradigm, with participants who watched a crime and then were asked to identify the culprit from a properly conducted identification procedure. Perhaps there are identification procedures that safeguard against phenotypic bias from biasing identification decisions and contaminating eyewitness accuracy.

### 1.2 Suspect bias versus general impairment in making eyewitness identification decisions

Scholars have posed two categories of factors that influence witness accuracy: general impairment and suspect-bias factors (Brewer and Wells, 2011; Smalarz, 2021). General impairment factors increase the likelihood that an eyewitness will make an identification error, but do not increase the rate of mistaken identifications of the suspect relative to identifications of known innocent lineup fillers. For example, errors caused by variables that increase the likelihood that the witness will make an identification (versus reject the lineup), such as failing to instruct the witness that the perpetrator may not be present in the lineup or presenting the lineup simultaneously (all photos at once) instead of sequentially (one photo at a time, no second lap), are expected to be distributed equally among lineup members, rather than being disproportionately directed to the suspect. The signal detection framework conceptualizes this willingness to make an identification of anyone as response bias (Wixted and Mickes, 2014).

In contrast, suspect bias factors encourage the witness to choose the suspect rather than any of the known-innocent fillers, meaning suspect identifications increase while filler identifications decrease (Kovera and Evelo, 2017; Smalarz, 2021). Factors that create suspect bias make suspects more vulnerable to identification (i.e., increased suspect identification rates) than they would be without that feature—regardless of whether the suspect is guilty or innocent. For example, when the lineup is biased such that the known-innocent fillers do not match the suspect’s appearance, the likelihood the eyewitness will choose the suspect increases relative to the choice of fillers (Fitzgerald et al., 2013). If a witness’s identification decision is driven by something other than their own memory for the culprit, the lineup should be deemed unfair, even in cases when the culprit is present in the lineup and the witness accurately identifies them, because the identifications are induced through suggestiveness and therefore are legally illegitimate hits (Wells et al., 2012). If this increase of both correct and incorrect identifications of suspects occurs in the absence of an increase in overall choosing (rejection rates remain unchanged), neither discriminability nor response criterion have changed. This pattern of effects was documented in the non-blind lineup administration literature (Kovera and Evelo, 2017), in which there is a reliable filler-to-suspect shift in identifications that reflects neither changes in discriminability nor changes in response criterion. When a lineup identification procedure is tainted by suspect bias, the signal detection framework does not provide appropriate analysis for identifying its prejudicial nature, which affects the due process of both innocent and guilty suspects.

However, little to no research directly tests whether general impairment factors will moderate the influence of suspect bias factors but not other general impairment factors (Brewer and Wells, 2011). Phenotypic bias functions as a general impairment factor in eyewitness identifications: more stereotypically Black faces facilitated erroneous feelings of familiarity and recognition errors, with stereotypically Black faces being *more* likely and less phenotypically African faces being *less* likely to be mistakenly identified as previously seen (Knuycky et al., 2013). Moreover, phenotypic bias (a general impairment factor) has the potential to interact with biased lineup composition (a suspect bias factor) in a unique way. As discussed above, a lineup procedure can be suspect-biased if the lineup composition does not adequately protect the suspect, such as in cases when the fillers do not match the suspect’s appearance (perhaps because the fillers do not share the same phenotypic facial features as the suspect). Suspect bias factors bias the witness toward choosing the suspect. General impairment factors may magnify this bias toward the suspect through a shift toward leniency in witnesses’ criteria to make a positive identification from the lineup, decreasing rejections of the lineup overall (Brewer and Wells, 2011).

However, consider a case in which a Black suspect is less phenotypically African than his surrounding lineup members. The phenotypic differences between the suspect’s and the fillers’ appearance should steer witnesses toward picking that suspect over other fillers, regardless of his guilt status. Yet, previous research on phenotypic bias suggests that witnesses may implicitly associate people with more African phenotypes with criminality (Eberhardt et al., 2004, 2006), causing them to identify the fillers with more African phenotypes over the suspect with a less Afrocentric phenotype. Rather than biasing witnesses toward identifying the suspect, a phenotypic mismatch between suspect and fillers may steer witnesses away from identifying a suspect with less phenotypically African features (despite matching the phenotypic expression of the witnessed culprit) in favor of one of the more Afrocentric looking fillers, due to phenotypic bias. In this case, biased lineup composition—traditionally considered a suspect bias factor (Smalarz, 2021)—may protect more Eurocentric suspects and bias witnesses toward choosing a known-innocent filler.

### 1.3 Current study: goals and research questions

To investigate whether there are procedural safeguards that can be used to reduce the potential effects of phenotypic bias on eyewitness identifications, we evaluated whether phenotypic bias interacts with lineup presentation and composition to affect suspect identifications and accuracy. When witnesses are tasked with making an identification from a lineup, they might identify the police’s suspect, which represents a correct *hit* in cases when the guilty culprit is present, and a problematic *false alarm* when the suspect is innocent (Cutler and Kovera, 2010). Alternatively, they might identify a known-innocent filler, which is a relatively harmless misidentification as the fillers will not be prosecuted, but is consistent with the witness using a more liberal response criterion (Cutler and Kovera, 2010; Lee and Penrod, 2019). Finally, witnesses might reject the lineup, which represents a *correct rejection* when the lineup contains an innocent suspect instead of the guilty culprit and a *miss* when the lineup contains the guilty culprit (Cutler and Kovera, 2010). The current study seeks to investigate racial bias and lineup fairness, rather than memory optimization, by observing whether a suspect’s phenotype in relation to the phenotype of the fillers creates suspect bias by disproportionately increasing the likelihood that a witness will identify them. To investigate suspect bias (Smalarz, 2021), we will report correct hits and false alarms collapsed into an encompassing suspect identification variable. To observe potential effects on accuracy, we will compare identification decisions across culprit-present and culprit-absent lineups. If an independent variable had a main effect on affects suspect identifications, yet failed to on its own but does not interact with the culprit-presence variable, then the independent variable increased correct it influences both accurate identifications of the culprit and mistaken inaccurate identifications of the innocent suspect at the same rate. If equal rates. Obtaining this pattern of effects were to obtain, then the results would indicate that discriminability of the culprit from the innocent suspects would be was unaffected, despite an increased likelihood of choosing the designated suspect (suspect bias; Smalarz, 2021). However, if the culprit-presence variable were to interact with an independent variable to influence suspect identification decisions, the independent variable would have affected discriminability as it was increasing or decreasing identifications of the culprit from culprit-present (guilty suspect) and culprit-absent (innocent suspect) at different rates (Wixted and Mickes, 2014).

Photographic lineups are typically presented in one of two ways: simultaneously, meaning all photos are visible to the witness at once, or sequentially, meaning each photo is presented one at a time, and the witness is asked to render a judgment on each photo before moving onto the next. Presenting a lineup sequentially reduces the rate at which witnesses mistakenly identify innocent suspects (Steblay et al., 2011). One explanation for this reduction in misidentifications is that sequential lineups prompt witnesses to make absolute judgments about whether a lineup member matches their memory for the perpetrator, whereas simultaneous lineups prompt witnesses to make relative judgments about which lineup member looks the most like the perpetrator, much like choosing the “best” answer on a multiple-choice test (Wells, 1984). In lineups that do not contain the perpetrator of the crime, simultaneous lineups pose a danger to innocent suspects who look the most like the perpetrator, relative to the other lineup members. Other scholars argue that the reduction in misidentifications seen in sequential lineups is caused by a higher degree of certainty required for witnesses to make an identification from a sequential lineup compared to a simultaneous lineup (Meissner et al., 2005; Flowe and Ebbesen, 2007; Goodsell et al., 2010). This type of criterion shift should affect the rate of mistaken choosing for both suspects and fillers by increasing or decreasing the criterion the witness sets for how well a face needs to match their memory for the culprit before choosing to identify them.

These interpretations of the sequential superiority effect have been challenged by proponents of diagnostic feature detection theory (DFDT), which predicts a memory advantage for simultaneous lineups compared to sequential lineups, because witnesses are better able to compare features of different lineup members when presented simultaneously (Wixted and Mickes, 2014). However, although responses tend to be more conservative for sequential lineups, some studies find little or no difference between the two procedures in underlying discriminability (Palmer and Brewer, 2012; Kaesler et al., 2020). Further, any differences in underlying discriminability between simultaneous and sequential lineups may simply reflect methodological choices that lack ecological validity (e.g., the first-yes-counts instruction; Horry et al., 2021; Winter et al., 2023). Because the current study’s focus is investigating suspect bias (e.g., the likelihood of witnesses identifying the designated suspect due to non-memorial factors), this study will not focus on discriminability.

#### 1.3.1 Hypotheses

If it is the case that general impairment factors will not interact to influence eyewitness accuracy, and both phenotypic bias and simultaneous presentation are general impairment factors, they should not interact to affect eyewitness decisions. Both factors should increase the likelihood that a witness chooses to identify someone from a lineup (reduce lineup rejections), but the increase should occur for both fillers and suspects if the lineup is fairly constructed. If so, sequential lineups will not serve as a safeguard against the general impairment caused by phenotypic bias. However, when the suspect has phenotypically African features that are not shared by the other lineup members, relative judgments made during simultaneous lineups may shift choosing toward that suspect. In this way, a general impairment factor (phenotypic bias) could act as a suspect biasing factor (unfair lineup composition). Because sequential lineups do not allow for this type of relative comparison, sequential lineup presentation should show less evidence of phenotypic bias when the lineup composition is unfair due to a mismatch between the phenotype of the suspect and the fillers. This prediction is consistent with findings that the superiority of the sequential procedure is maximized when the suspect is distinctive (Carlson et al., 2008) and that sequential presentation reduces other forms of bias (Lindsay et al., 1991).

To test these propositions, we conducted an experiment in which we manipulated the phenotype of the culprit, whether the fillers in a photo-array matched the suspect’s phenotype, whether the photo-array was conducted simultaneously or sequentially, and whether the photo-array was culprit-absent or -present. The phenotype of innocent suspects in culprit-absent lineups always matched the phenotype of the witnessed culprit. We predicted that suspect phenotype would produce a main effect, with witnesses more likely to identify the suspect when the suspect had features that were more, rather than less, Afrocentric (hypothesis 1). We also predicted that presentation method would produce a main effect, with witnesses more likely to identify the suspect when the lineup was presented simultaneously compared to sequentially (hypothesis 2). Finally, we predicted a three-way interaction between phenotypic bias, lineup construction, and lineup presentation style (hypothesis 3). Specifically, we hypothesized that when lineups were presented simultaneously, there would be a significant two-way interaction of phenotypic bias and lineup composition, such that the simple main effect of phenotypic bias will be greater when lineups were suspect-biased (i.e., the fillers are a phenotypic mismatch to the suspect) than when all lineup members shared features with the same phenotype (hypothesis 3a). We expected that this interaction would be significantly smaller or non-significant for sequential lineups (hypothesis 3b).

## 2 Materials and methods

To test these predictions, we conducted an experiment with a 2 (Culprit/Innocent Suspect Phenotype: More African vs. Less African) × 2 (Lineup Fillers: Phenotypic Match to Culprit/Innocent Suspect vs. Phenotypic Mismatch) × 2 (Photo Presentation: Simultaneous vs. Sequential) × 2 (Array Type: Culprit-present vs. Culprit-absent) between-subjects factorial design. Our dependent variables of interest were the witness’s identification decision and their confidence in that decision.

### 2.1 Participants

Because there are no statistical packages for reliably determining needed sample sizes to power for a three-way interaction effect within a logistic regression analysis (Aberson, 2019), we approached estimating the sample size we needed to sufficiently power the test of our predicted effects in two ways. First, we conducted a power analysis for the test of the predicted three-way interaction using an ANOVA with GPower 3.1.9.1 (Faul et al., 2009). That analyses suggested that a sample of 560 participants would be sufficient to detect relatively small effects (partial η^2^ = 0.02) with alpha = 0.05 and power = 0.80, even after taking into account that power is multiplicative across our three predicted effects (Maxwell, 2004; Schimmack, 2012). Second, we compared this suggested sample size with crude estimates for sufficient sample size in logistic regression models, including having at least 10 cases per predictor with a sample size of 500 typically producing adequate power (Long, 1997). In our analyses, there were at most 11 predictors (4 main effects, 6 two-way interactions, and 1 three-way interaction) in the model.

Thus, we recruited 600 White adults (50% women, 3% White Hispanic) through Qualtrics Panels, which was the maximum number of participants we could recruit given our funding constraints. Participants ranged in age from 18 to 87 (*M* = 43.94, SD = 14.25). Participants were compensated $1.50 for their time.

### 2.2 Materials

Materials included the mock theft video, the photo arrays, and a Qualtrics questionnaire.

#### 2.2.1 Mock theft video

Participants viewed a video of a mock theft in which a Black man entered an office and stole an iPhone from a backpack located in that office. The culprit’s face was visible for 8 seconds. For purposes of stimulus sampling to increase the generalizability of our results (Wells and Windschitl, 1999), we video recorded six different versions of the theft, with a different actor portraying the culprit in each version. The videos are available to view online at https://osf.io/am5qh/?view_only=7cf96a56de9946348e001ef2885df2d7.

#### 2.2.2 Culprit phenotype

To ensure that the culprits were good representations of the categories they were to represent (e.g., Black men with facial features that were representative of a more or less African facial phenotype), graduate students rated headshots of 86 Black men who responded to a solicitation on Craigslist.org to play a perpetrator in a mock crime video. Students rated the headshots on the actors’ attractiveness and distinctiveness on 1 (*not at all*) to 7 (*extremely*) Likert-type scales. They also rated the extent to which the actors looked stereotypically Black on a 1 (*not at all*) to 9 (*extremely*) Likert-type scale. They rated age on a 10-point scale, with each point representing a 5-year increment, starting with 15–20 and ending with 60+. Finally, they also indicated the perceived race of the actors. We selected six actors, three of whom received low stereotypicality scores (mean scores ranging from 3.5 to 4.4) for use in our Less African condition) and three of whom received high stereotypicality scores (mean scores ranging from 6.2 to 6.8) for use in our More African condition. All six actors were correctly categorized as Black by over 90% of the pilot participants and were perceived as being between 20 and 40 years old by over 85% of participants. The actors were rated similarly on attractiveness, distinctiveness, and age. When we filmed the stimulus materials, we took photographs of the perpetrators displayed against a white background for later use in the culprit-present photo arrays.

#### 2.2.3 Photo array

We created six-person photo arrays for each culprit, each containing one suspect (either the culprit or a designated innocent suspect) and five fillers. The photo arrays orthogonally varied whether the culprit was present and whether the fillers matched the suspect’s phenotype. Fillers and innocent suspects were generated from a pool of 127 photos of Black men in the Chicago Face Database (Ma et al., 2015) or used in previous research (Eberhardt et al., 2004). All photographs were edited to depict the fillers’ and innocent suspects’ faces in front of a white background. Faces did not have facial hair or other distinctive features (i.e., unique hair styles, piercings, and tattoos). We also ensured that the fillers matched the characteristics most frequently mentioned in descriptions of culprits obtained from MTurk workers (240 workers provided descriptions, with 12 workers providing descriptions of each face in our facial database).

We selected one photograph from the facial databases to serve as the innocent suspect for each of the culprit-absent lineups (three innocent suspects in total). Similarly, we selected fillers for both the culprit-present and -absent lineups from these two databases. MTurk workers rated the faces distinctiveness, attractiveness, age, and stereotypicality and categorized the faces based on perceived race, with 15–22 participants rating each face. Participants correctly categorized all faces chosen to serve as innocent suspects and fillers as Black. Faces chosen to serve as innocent suspects matched the culprit on phenotype, attractiveness, distinctiveness, and age in that the 95% confidence interval (CI) of the mean difference between the rating of the culprit and of the innocent suspect included zero for attractiveness, distinctiveness, and age. The selected fillers had ratings of average distinctiveness (defined as ±2 SD of filler distinctiveness mean, *M* = 3.94, SD = 0.43), average attractiveness (±2 SD of filler attractiveness mean, *M* = 3.37, SD = 0.54), and an age similar to the culprits (*M* = 3.08, SD = 1.10). Fillers selected to serve as more African phenotypic fillers had stereotypicality ratings of 5.36–7.45 (*M* = 6.47, SD = 0.62). Fillers selected to serve as less African phenotypic fillers had stereotypicality ratings of 2.41–6.06 (*M* = 4.37, SD = 0.77). All 24 photo-arrays are available at https://osf.io/am5qh/?view_only=7cf96a56de9946348e001ef2885df2d7.

### 2.3 Procedure

This study was approved by the Institutional Review Board at John Jay College of Criminal Justice (City University of New York) and run online using Qualtrics. After consenting to participate and providing their demographic information, participants were given the following instructions, “You are going to be shown a short film. Pay close attention because you will be asked some questions afterwards. There will be no audio in this film. When you are ready to view the film, please press the NEXT button.” On the next page of the survey, participants were provided further instructions, which were displayed above the simulated crime video and read, “Please watch the video. There is no sound. Do not click or pause the video. You can advance this page once the timer is finished. The page will automatically advance after 4 minutes.” Each sentence in both instructions was displayed as a separate bullet point. Participants watched one version of the mock crime video, in which they viewed one of our Black male culprits who appeared either more phenotypically African or less phenotypically African. After viewing the crime video, participants were instructed, “What you just witnessed was a crime recorded on video surveillance. In a few minutes, you will be asked to identify the individual who stole the phone in the video. Before that occurs, we want to measure your attention”. Participants were then instructed on and completed a 3-min word puzzle as a filler task. Following the filler task, participants were provided lineup instructions. All participants were instructed, “Time: You may spend as long as you want on this page. You must spend at least 1 minute (see timer) to ensure comprehension.”

Participants in the simultaneous lineup conditions were instructed, “On the next page you will view six photos. Your task is to identify which photo, if any, depicts the culprit from the video who stole the phone. There is no time limit for this task. You may take as long as you like to make a decision and you may change your selection as many times as you like before you submit. Once you submit your answer (by clicking NEXT) you will not be able to return to the lineup. You should know: (1) The culprit might not be in the lineup at all, so the correct answer might be ‘not present,’ (2) If you feel unable to make a decision, you have the option of responding ‘don’t know,’ (3) After making a decision you will be asked to state how confident you are in that decision, (4) The investigation will continue if no identification is made.” On the next page displayed above the lineup, participants in the simultaneous lineup conditions were further instructed, “Below are photos of six faces. Please identify which photo depicts the individual who stole the phone in the video. If you do not think any of these photos depicts the person who stole the phone, please select ‘The culprit is not present.’ If you are unsure if the culprit is present or not, please select ‘I don’t know if the culprit is present.’ You will be asked to give your confidence in this decision on the next page.”

Participants in the sequential lineup conditions were instructed: “On the following pages you will view photos of several individuals. Each photo will be shown one at a time. Your task is to decide if the photo is of the same individual who stole the smart phone in the video you watched. For each photo, select YES if you do think that individual in the photo is the person who stole the smart phone. Select NO if you do not think that the individual in the photo is the person who stole the smart phone. There is no time limit for this task. You may take as long as you want on each page and you may change your selection (YES/NO) as many times as you like before you submit. Once you submit your answer (by clicking NEXT), you will not be able to go back. You will only be able to see each photo once. You should know: (1) The culprit might not be in the lineup at all, so the correct answer might be to choose NO for all photos, (2) After making a decision you will be asked to state how confident you are in that decision, (3) The investigation will continue even if no identification is made.” On the next pages displayed above each photo, participants in the simultaneous lineup conditions were further instructed, “Please decide if the photo below depicts the culprit from the video who stole the phone.”

Participants were presented one six-person lineup that contained either the culprit they saw in the video (culprit-present) or an innocent suspect (culprit-absent) who always matched the culprit’s phenotype, and had been rated as equally attractive, equally distinct, and around the same age (rating and selection procedure described above). The suspect in the lineup always matched the phenotype of the culprit witnesses viewed in the video (i.e., if a witness viewed a less Afrocentric perpetrator in the video, they were presented with a less Afrocentric suspect in the lineup, and if a witness viewed a more Afrocentric perpetrator in the video, they were presented with a more Afrocentric suspect in the lineup). The lineups also contained fillers who either matched the culprit/suspect’s phenotype (Phenotypic Match: less Afrocentric suspects/culprits surrounded by less Afrocentric fillers; more Afrocentric suspects/culprits surrounded by more Afrocentric fillers) or did not match the culprit/suspect’s phenotype (Phenotypic Mismatch: less Afrocentric suspects/culprits surrounded by more Afrocentric fillers; more Afrocentric suspects/culprits surrounded by less Afrocentric fillers). The suspect was always in position number five.

Lineups were either presented simultaneously (all photos at once) or sequentially (one at a time). Sequential lineups presented each photo in order one at a time and were concluded once the witness made an identification (first identification stopping rule). For each photo, participants in the sequential conditions answered “Yes” or “No” and were not given a “Don’t Know” response option. If participants went through all the photograph options without identifying anyone, their identification decision was recorded as a lineup rejection. Lineup photos were only presented once. Participant-witnesses in the simultaneous lineup conditions were instructed to either identify one of the six photos as the culprit, report that the culprit is not present, or indicate they do not know if the culprit is present. Not present and do not know responses were categorized as lineup rejections (i.e., non-identifications). Finally, participants reported their confidence in their identification decision (0%–100%) and completed a demographics questionnaire that asked for their age, gender, race/ethnicity, and education level.

## 3 Results

Participants’ identification decisions were re-categorized into three dichotomous variables: (1) suspect identification versus other (other = filler identifications and rejections), (2) filler identifications versus other (other = suspect identifications and rejections), and (3) rejections versus other (other = filler and suspect identifications). These variables allow us to investigate suspect bias, our main dependent variable of interest, while using culprit presence to detect any differences between accurate and inaccurate witnesses (where suspect identifications are correct *hits* in culprit-present lineups, but *false alarms* in culprit-absent lineups, and rejections are *correct rejections* in culprit-absent lineups but *misses* in culprit-present lineups). Identification decisions across experimental conditions are presented in Table 1. Measures of discriminability (*d’*) and response bias (*c*) for each experimental condition are available in the supplemental materials (Supplementary Table 1).

**TABLE 1:** Frequency of identification decisions in each condition.

Independent variables										
Culprit/suspect features	Lineup composition	Presentation	Array type	Suspect		Filler		Rejections		Total
				*n*	%	*n*	%	*n*	%	*n*
More phenotypic	Mismatch	Simultaneous	Present	16	41	10	26	13	33	39
			Absent	6	19	8	26	17	55	31
			Total	22	31	18	26	30	43	70
		Sequential	Present	10	28	14	39	12	33	36
			Absent	4	9	18	41	22	50	44
			Total	14	18	32	40	34	43	80
		Total		36	24	50	33	64	43	150
	Match	Simultaneous	Present	10	29	14	40	11	31	35
			Absent	1	3	14	39	21	58	36
			Total	11	15	28	39	32	45	71
		Sequential	Present	4	10	25	63	11	28	40
			Absent	1	3	19	49	19	49	39
			Total	5	6	44	56	30	38	79
		Total		16	11	72	48	62	41	150
	Total			52	17	122	41	126	42	300
Less phenotypic	Mismatch	Simultaneous	Present	11	29	10	26	17	45	38
			Absent	5	13	11	29	22	58	38
			Total	16	21	21	28	39	51	76
		Sequential	Present	7	19	11	30	19	51	37
			Absent	8	22	12	32	17	46	37
			Total	15	20	23	31	36	49	74
		Total		31	21	44	29	75	50	150
	Match	Simultaneous	Present	11	24	15	33	20	43	46
			Absent	4	11	16	43	17	46	37
			Total	15	18	31	37	37	45	83
		Sequential	Present	6	21	13	45	10	35	29
			Absent	1	3	18	47	19	50	38
			Total	7	10	31	46	29	43	67
		Total		22	15	62	41	66	44	150
	Total			53	18	106	35	141	47	300
Total				105	18	228	38	267	45	600

Values are given in raw counts and percentages within conditions. Suspect identifications are correct hits in culprit-present lineups and false alarms in culprit-absent lineups. Rejections are correct rejections in culprit-absent lineups and misses in culprit-present lineups. Rejections include both “not present” and “don’t know” responses. The percentages in some rows do not total 100 because of rounding.

We used IBM’s SPSS Version 28 to conduct forced-entry, hierarchal binary logistic regressions to test our predicted effects on our three dependent variables: suspect identifications, filler identifications, and photo array rejections. We entered suspect/culprit phenotype, lineup presentation style, filler composition, and culprit-presence as predictors in the first block, all two-way interactions into the second block, and our predicted three-way interaction between phenotypic bias, lineup construction, and lineup presentation into the third block. We ran analyses twice: once including all participants, both White Hispanics (*n* = 18, 3%) and White Europeans (*n* = 582, 97%), and once excluding White Hispanic participants and only including White European participants. Excluding White Hispanics did not produce significant differences in the pattern of the results, so we have reported the results based on the data provided by both White Europeans and White Hispanics.

This study was designed and powered to evaluate identification decision rates, but participant-witnesses also reported their numeric confidence in their identification decision. Our confidence analysis is exploratory, as we did not make prior predictions regarding differences in confidence nor collect enough observations to appropriately power for confidence-accuracy analysis. For our exploratory analysis, we ran an analysis of variance using IBM’s SPSS Version 28 general linear model univariate procedure to compare confidence means across groups. Confidence-accuracy characteristic (CAC) analysis comparing the accuracy of highly confident suspect identifications in the phenotypic match versus phenotypic mismatch conditions was calculated using Microsoft Excel and plotted using R Version 4.3.1 ggplot package. Results of our confidence analyses are available in Supplementary Tables 2–5 and Supplementary Figure 1.

### 3.1 Suspect identification rates

Using suspect identifications as our dependent variable, we entered suspect/culprit phenotype, lineup presentation style, filler composition, and culprit-presence as predictors in the first block, all two-way interactions into the second block, and our predicted three-way interaction between suspect/culprit phenotype, lineup presentation style, and filler composition into the third block. The second block of the analyses did not show significant improvement in the model, Wald’s χ^2^ (6, *N* = 600) = 9.36, *p* = 0.155, nor did the third block, Wald’s χ^2^ (1, *N* = 600) = 0.01, *p* = 0.946. Because our interaction terms did not improve model fit, we report results from the first block below in-text (Field, 2013). The full-factorial model is presented in Supplementary Table 6. All two-way interactions and our predicted three-way interaction between suspect/culprit phenotype, lineup presentation style, and filler composition were not statistically significant, and the main effects for lineup composition and lineup presentation reported below lose significance when interaction terms are included.

**FIGURE 1:**
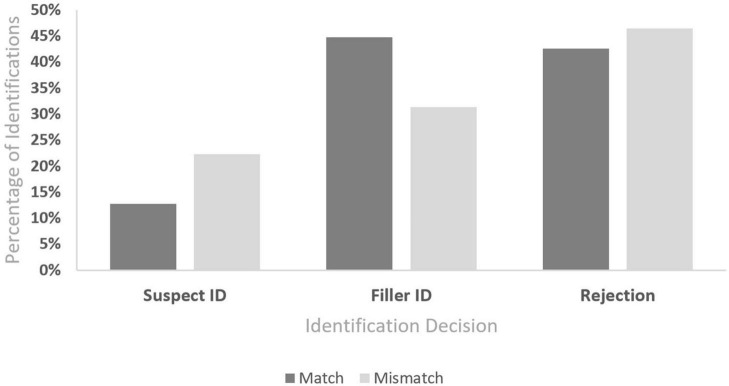
Identification decisions by lineup compositions.

Contrary to our first hypothesis, there was no main effect of phenotypic bias. Witnesses were no more likely to identify the suspect when the suspect had more Afrocentric features (17%) than when he had less Afrocentric features (18%), *B* = 0.005, SE = 0.22, Wald’s χ^2^ (1, *N* = 600) = 0.001, *p* = 0.982, OR = 1.01, 95% CI [0.65, 1.56]. However, the phenotypic match of the lineup fillers to the suspect produced a significant main effect, such that witnesses were more likely to identify the suspect when the other lineup fillers did not match the suspect’s phenotype (Figure 1). Witnesses who were presented with a suspect with relatively more Afrocentric features surrounded by fillers with relatively less Afrocentric (more Eurocentric) features or a suspect with relatively less Afrocentric features surrounded by fillers with more Afrocentric features were more likely to identify the mismatched suspect (22%) than were witnesses who were presented with a lineup composed of faces that all matched in phenotype (13%), *B* = 0.73, SE = 0.23, Wald’s χ^2^ (1, *N* = 600) = 10.35, *p* = 0.001, OR = 2.08, 95% CI [1.33, 3.26], irrespective of whether that phenotype was more or less African.

We found support for our second hypothesis, as there was a significant main effect for lineup presentation, such that witnesses were more likely to identify the suspect when the lineup was presented simultaneously (21%) compared to sequentially (14%), *B* = −0.53, SE = 0.23, Wald’s χ^2^ (1, *N* = 600) = 5.53, *p* = 0.019, 1/OR = 1.70, 95% CI [1.09, 2.65]. This analysis also reproduced the well-established finding that participant-witnesses make more identifications of guilty culprits from culprit-present lineups (25%) than identifications of innocent suspects from culprit-absent lineups (10%), *B* = 1.10, SE = 0.24, Wald’s χ^2^ (1, *N* = 600) = 21.5, *p* < 0.001, OR = 3.00, 95% CI [1.82, 4.78]. Despite no significant interaction emerging between lineup presentation and culprit presence, a closer look at the data reveals that the increase in suspect identifications in simultaneous lineups was driven by choosing in culprit-present lineups: participant-witnesses were more likely to accurately identify the guilty culprit in simultaneous lineups compared to sequential lineups (30% vs. 19%), but nearly equally likely to inaccurately misidentify the innocent suspect (11% vs. 9%).

### 3.2 Filler identifications

Using filler identifications as our dependent variable, we entered suspect/culprit phenotype, lineup presentation style, filler composition, and culprit-presence as predictors in the first block, all two-way interactions into the second block, and our predicted three-way interaction between suspect/culprit phenotype, lineup presentation style, and filler composition into the third block. The second block of the analyses did not show significant improvement in the model, Wald’s χ^2^ (6, *N* = 600) = 2.96, *p* = 0.814, nor did the third block, Wald’s χ^2^ (1, *N* = 600) = 0.07, *p* = 0.791. Because our interaction terms did not improve model fit, we report results from the first block below in-text (Field, 2013). The full-factorial model is presented in Supplementary Table 7. All two-way interactions and our predicted three-way interaction between suspect/culprit phenotype, lineup presentation style, and filler composition were not statistically significant, and the main effects for lineup composition and lineup presentation reported below lose significance when interaction terms are included.

Again, there was no main effect of phenotypic bias. Witnesses were not significantly more likely to identify a known-innocent filler when the suspect had more stereotypically African features (41%) than less stereotypically African features (35%), *B* = 0.21, SE = 0.17, Wald’s χ^2^ (1, *N* = 600) = 1.43, *p* = 0.232, OR = 1.23, 95% CI [0.88, 1.72]. The manipulation of the phenotypic match of the fillers to the suspects produced a significant main effect, *B* = −0.59, SE = 0.17, Wald’s χ^2^ (1, *N* = 600) = 11.86, *p* < 0.001, 1/OR = 1.81, 95% CI [1.29, 2.53]. Witnesses were more likely to identify a known-innocent filler when the lineup fillers matched the suspect’s phenotype (Figure 1). Witnesses who were presented with a suspect with more Afrocentric features surrounded by fillers with less Afrocentric (more Eurocentric) features or a suspect with less Afrocentric features surrounded by fillers with more Afrocentric features were less likely to identify a known-innocent filler (31%) than were witnesses who were presented with a lineup composed of faces that all matched in phenotype (45%).

There was a significant effect of lineup presentation such that witnesses were more likely to identify a known-innocent filler when the lineup was presented sequentially (43%) as opposed to simultaneously (33%), *B* = 0.47, SE = 0.17, Wald’s χ^2^ (1, *N* = 600) = 7.36, *p* = 0.007, OR = 1.6, 95% CI [1.14, 2.24]. Finally, participant-witnesses were no more likely to make filler identifications from culprit-absent lineups (39%) than they were from culprit-present lineups (37%), *B* = −0.03, SE = 0.17, Wald’s χ^2^ (1, *N* = 600) = 0.04, *p* = 0.846, 1/OR = 1.03, 95% CI [0.74, 1.45].

### 3.3 Lineup rejections

Using lineup rejections as our dependent variable, we entered suspect/culprit phenotype, lineup presentation style, filler composition, and culprit-presence as predictors in the first block, all two-way interactions into the second block, and our predicted three-way interaction between suspect/culprit phenotype, lineup presentation style, and filler composition into the third block. As mentioned above, our “lineup rejections” variable includes both “not present” and “don’t know” responses. The second block of the analyses did not show significant improvement in the model, Wald’s χ^2^ (6, *N* = 600) = 4.86, *p* = 0.563, nor did the third block, Wald’s χ^2^ (1, *N* = 600) = 0.09, *p* = 0.760. Because our interaction terms did not improve model fit, we report results from the first block below (Field, 2013), along with one significant non-predicted two-way interaction that emerged in the second block and remained significant in the third block. The full-factorial model is presented in Supplementary Table 8. All other two-ways and our predicted three-way interaction are not statistically significant, and the main effect for culprit-presence reported below loses significance when interaction terms are included.

Once again, there was no main effect of suspect phenotype. Witnesses were not significantly more likely to reject the lineup when the suspect had more Afrocentric features (42%) than when he had less Afrocentric (more Eurocentric) features (47%), *B* = −0.20, SE = 0.17, Wald’s χ^2^ (1, *N* = 600) = 1.42, *p* = 0.234, 1/OR = 1.22, 95% CI [0.88, 1.69].

In contrast to its effects on suspect and filler identifications, whether the phenotype of the fillers matched that of the suspect did not affect the rate of lineup rejections. Witnesses were no more likely to reject the lineup when the suspect’s phenotype mismatched the fillers’ phenotype (46%) than when the suspect’s phenotype matched the fillers’ phenotype (43%), *B* = 0.16, SE = 0.17, Wald’s χ^2^ (1, *N* = 600) = 0.88, *p* = 0.348, OR = 1.20, 95% CI [0.85, 1.62] (Figure 1). Similarly, lineup presentation did not affect the rate of lineup rejections; witnesses were equally likely to reject the lineup when the lineup was presented sequentially (43%) as opposed to simultaneously (46%), *B* = −0.15, SE = 0.17, Wald’s χ^2^ (1, *N* = 600) = 0.78, *p* = 0.377, OR = 0.86, 95% CI [0.62, 1.20].

Participant-witnesses were more likely to reject the lineup when the culprit was absent (51%) than when the culprit was present (37%), *B* = −0.57, SE = 0.17, Wald’s χ^2^ (1, *N* = 600) = 11.60, *p* < 0.001, 1/OR = 1.76, 95% CI [1.27, 2.44]. This main effect was qualified by a significant two-way interaction that emerged between suspect phenotype and culprit presence, *B* = −0.66, SE = 0.34, Wald’s χ^2^ (1, *N* = 600) = 3.83, *p* = 0.050, 1/OR = 1.93, 95% CI [1.00, 3.73]. Although correct rejection rates were about equal when the lineup contained an innocent suspect (more Afrocentric = 53%, less Afrocentric = 50%), participant-witnesses were more likely to inaccurately reject a culprit-present lineup when the guilty suspect had more Eurocentric features (44% incorrect rejection) than when the guilty suspect had more Afrocentric features (31% incorrect rejection).

## 4 Discussion

There are large racial disparities in the number of wrongful convictions based on eyewitness misidentifications of Black versus White defendants. The size of the own-race bias effects is not sufficient to explain these disparities (Katzman and Kovera, 2023). Although some of these disparities may be the result of disparate policing practices that lead more innocent Black than White men to be subjected to the risk of misidentification (Katzman and Kovera, 2023), there may be other racial biases that contribute to them. Phenotypic bias, a bias against individuals who have more Afrocentric facial features (Knuycky et al., 2013), may help to explain this disparity. Because phenotypic bias operates on Black rather than White target lineups, it is possible that it may put innocent Black suspects at greater risk of misidentification. This experiment had three goals: (a) to examine whether phenotypic bias affects eyewitness identification decisions, (b) to investigate whether a phenotypic mismatch between fillers and a suspect may bias the lineup against the suspect, and (c) to explore whether sequential lineup presentation might guard against the harmful effects of phenotypic bias and phenotypic mismatch.

### 4.1 Suspect phenotype effects

Phenotypic bias did not influence our participant-witnesses’ identification decisions in the way that we hypothesized. Indeed, we found little evidence that witnesses were more likely to identify suspects if they had more Afrocentric features rather than more Eurocentric features, whether they were the culprit or an innocent suspect. The only evidence to support the supposition that a more African phenotype promotes mistaken identifications comes from our finding that participants were more likely to incorrectly reject a culprit-present lineup when the guilty suspect had less stereotypically African features than when the guilty suspect had more stereotypically African features. This finding suggests that our participants used a higher criterion for identifying the less Afrocentric culprit than the more Afrocentric culprit. However, the increased choosing rates for more Afrocentric suspects were distributed evenly across both suspects and fillers, and thus did not differentially increase suspect identifications in this study. Further, this finding was unexpected, obtained from a logistic regression model that did not improve model fit, and was significant at *p* = 0.050, all of which suggest that this finding should be interpreted with caution. Other than this one effect, phenotype—on its own—had little influence on witnesses’ decisions.

There are several possibilities for why phenotypic bias failed to appear in this eyewitness context. First, the type of phenotypic bias observed in other research may simply not extend to eyewitness recognition tasks. In previous studies, phenotypic bias affected inferences about criminality (Eberhardt et al., 2004; Kahn and Davies, 2011) and deservingness of punishment (Blair et al., 2004b; Eberhardt et al., 2006). These types of inferences may be more susceptible to bias than a facial recognition task, in which witnesses’ judgments are at least somewhat constrained by their memory for the culprit and whether any of the photos before them provide a good match to their memory (Clark, 2003). However, we also may have simply failed to produce phenotypic bias because of our study design. In all our lineups, the suspect matched the culprit’s phenotype. Future research should manipulate phenotypic match between the culprit and the suspect to investigate whether witnesses are more likely to misidentify an innocent suspect with more Afrocentric features when the culprit had more Eurocentric features than when the reverse is true (i.e., an innocent person with more Eurocentric features is suspected of being a culprit who had more Afrocentric features).

It is also possible that phenotypic bias would have extended to eyewitness recognition tasks in the past, but increased societal attention to implicit racial bias provided our participants with the self-awareness and motivation to avoid acting on these biases. With the massive boom in the Black Lives Matter movement after the murder of George Floyd in 2020, experimental work on racial bias has diverged from real-world field data which consistently demonstrates racial bias, in part due to social desirability effects (Salerno et al., 2023; Smalarz et al., 2023). This explanation is somewhat less likely as the data were collected before the COVID-19 pandemic began, thus before the racial unrest prevalent in the aftermath of Floyd’s murder. However, the possibility that social desirability concerns were present prior to these events remains.

### 4.2 Lineup composition effects

Even though our participants did not exhibit phenotypic bias in their identifications of suspects, they were sensitive to variations of phenotype among the people depicted in the photo arrays. Participant identification decisions were affected by whether the suspect had a different phenotypic expression than the known-innocent fillers. When there was a phenotypic mismatch between the suspect and the lineup fillers, witnesses were more likely to identify the suspect, regardless of whether the suspect was guilty. That is, witnesses were (a) more likely to identify the suspect from a biased rather than an unbiased photo array (b) less likely to identify fillers from a suspect biased photo array, and (c) no more likely to state that a culprit is not present from a biased than unbiased photo array (Figure 1). This pattern of results resembles findings from the double-blind administration literature, known as the “filler-to-suspect shift” (Kovera and Evelo, 2017). The filler-to-suspect shift represents the phenomenon that when a lineup administration is single-blind (i.e., when the lineup administrator knows the identity of the suspect), the witness is more likely to identify the suspect and less likely to identify a filler than when the lineup administrator is double-blind (i.e., when the lineup administrator does *not* know the identity of the suspect). However, administrator knowledge does not affect the likelihood that the witness rejects the lineup. Because administrator knowledge of the suspect does not increase the proportion of witnesses who make an identification, administrator knowledge does not affect witnesses’ criterion to make an identification (Kovera and Evelo, 2017). Thus, just as the non-blind administrator communicates the identity of the suspect to the witness, our mismatched phenotypic lineups communicated to witnesses which photo depicted the suspect, especially to witnesses who were willing to identify someone from the photo array but may not have a strong match between their memory of the culprit and any particular member of the lineup. We observed no shift in decision criterion to make an identification, as rejection rates were the same across phenotypically matched lineups and phenotypically mismatched lineups. However, their ability to discern which lineup member is the suspect among the fillers increased in mismatched lineups (suspect bias). This increased discernment of the suspect did not translate into the ability to discriminate guilty suspects from innocent suspects; instead, it simply created suspect bias, rather than improving signal discriminability versus noise. The phenotypic mismatch of the suspect with the fillers leads those willing witnesses to choose the mismatched suspect rather than a filler. Thus, it may be particularly important to ensure that fillers match suspects on phenotypic expression when witness memories are weaker or when their criterion for choosing may be low.

Our findings underscore the theoretical importance of examining the variety of ways that suspect bias manifests (Smalarz, 2021) and the methodological importance of designating an innocent suspect in culprit-absent lineups, as these analyses would not have been possible otherwise. Put simply, mismatched phenotypes can make the suspect “stand out” in the lineup and put innocent suspects at greater risk of misidentification. A large body of research has investigated biasing factors of lineups, including mismatched backgrounds, clothing, and lighting (Lindsay et al., 1987; Harvard et al., 2023). Indeed, witnesses in real cases are more likely to identify suspects when the lineup is demonstrably biased toward the suspect according to mock witness studies of lineup fairness (Steblay and Wells, 2020). Yet phenotypic mismatching has escaped empirical notice.

Overall, to reduce disproportionate identifications of Black suspects, lineup fillers should always match the suspect’s phenotype. However, an archival study of 250 offender descriptions by witnesses of armed bank robberies revealed that when describing the offender, witnesses reported few identifying details and information related to phenotype was not among the frequent descriptors used (Fahsing et al., 2004). One method that police officers use to construct lineups is to find known-innocent fillers who match the witness’s description of the culprit (e.g., build, hair, and race). However, if phenotype expression is not included in these descriptions, fillers who match the suspect on every other descriptor will still not provide adequate protection for the suspect. When investigators rely on witnesses’ imprecise descriptions to construct their photo arrays, known-innocent fillers that match the general description of the culprit provided by the witness may possess disqualifying features that ultimately reduce the lineup’s functional size and the protections provided to the suspect. Researchers are developing interview strategies to elicit bountiful and accurate offender descriptions from witnesses. For example, the person description interview (PDI), which includes a general-to-specific instruction (GSI) and a down-to-up instruction (DUI) tested both in the laboratory and in the field, meaningfully increased the amount and accuracy of facial descriptors (Demarchi and Py, 2009). If a witness’s memory for the perpetrator is not strong enough to provide a detailed description, the reliability of any positive identification they make should be questioned.

### 4.3 Lineup presentation effects

Participants’ identification decisions were influenced by how the lineup was presented. When the lineup was presented simultaneously rather than sequentially, participants were more likely to identify the suspect, less likely to identify a known-innocent filler, and equally likely to reject the lineup. However, lineup presentation was included in our study design for its potential to mitigate the problematic effects of both phenotypic bias and phenotypic mismatch in lineup composition. Although we did not find an effect for phenotypic bias (and thus no intervention is required to address it), sequential presentation failed to protect innocent suspects: a closer look at our data revealed that the increase in suspect identifications we observed in simultaneous lineups was driven by choosing in culprit-present lineups, such that participant-witnesses were much more likely to accurately identify the guilty culprit in simultaneous lineups compared to sequential lineups (30% vs. 19%), but nearly equally likely to inaccurately misidentify the innocent suspect (11% vs. 9%).

This pattern of results partially mirrors findings from other studies in which witnesses were more likely to positively identify perpetrators from culprit-present lineups presented simultaneously rather than sequentially (Steblay et al., 2001, 2011; Steblay and Wells, 2020). However, meta-analyses also find that witnesses are more likely to correctly reject lineups from culprit-absent sequential than culprit-absent simultaneous lineups (Steblay et al., 2001, 2011). The current study did not reproduce this effect: participant-witnesses correctly rejected the culprit-absent lineup 54% of the time when it was presented simultaneously, and 49% of the time when presented sequentially. Instead, our participant-witnesses were more likely to make a filler identification from sequential lineups (culprit-present = 44%, culprit-absent = 42%), than from simultaneous lineups (culprit-present = 31%, culprit-absent = 35%). Thus, although suspect identification rates were higher overall in simultaneous lineups, sequential lineup presentation did not provide protections for innocent suspects, and only acted to reduce accurate culprit identifications.

Although scholars argue and there is empirical evidence that sequential presentation can reduce mistaken identifications resulting from suspect bias in photo arrays, perhaps by diminishing eyewitnesses’ reliance on relative judgment processes (Lindsay and Wells, 1985), we found that sequential lineup presentation was an inadequate safeguard for suspect bias based on phenotypic mismatch. Perhaps the strength of our manipulation of phenotypic mismatch was strong enough and noticeable enough to allow witnesses to hold that information in mind when making their decisions about sequentially presented photos. Whatever the reason, given that phenotypic match seems to operate differently than other types of suspect bias, it is ripe for continued empirical examination.

## 5 Future research and conclusion

This study was conducted entirely online. Although we took care to maximize the study’s ecological validity by filming a realistic mock crime video and including a filler task, the social context in which identifications are made can influence the identifications made by witnesses (Kovera and Evelo, 2021). Future researchers could benefit from exploring these questions using in-person paradigms. Additionally, the filler task in this study only provided a 3-min retention interval between viewing the perpetrator and being asked to make an identification. Ecological validity would be heightened if future researchers use a retention interval that more accurately matches the average interval witnesses experience in the field. In addition, to provide better recommendations to law enforcement, future research could tease apart which of these prototypically African features witnesses rely on most by isolating and manipulating each feature. For example, perhaps fillers need only match the suspect on skin tone and hair texture, but not nose shape.

Future research should also examine the generalizability of these effects to contexts in which the encoding conditions are more favorable to witness memory. To explore whether phenotypic bias affects eyewitness identification decisions, we intentionally created encoding conditions (e.g., an 8 s exposure duration) that were likely to produce weak memory traces that would allow for bias to operate. As a result, we obtained more lineup rejections and fewer suspect identifications than are typically seen in actual eyewitness identification decisions (see Wells et al., 2020 for a review of estimates of the types of eyewitness decisions made by witnesses in actual cases). Scholars should explore the extent to which these findings hold under better encoding conditions.

Moreover, this study was not designed to investigate the role phenotypic bias plays on the *own-race bias* (ORB), as we only investigated White participant-witnesses attempting identifications of Black perpetrators from lineups composed entirely of Black men. Although empirical studies consistently produce the ORB, there is substantial (and currently inexplicable) variation in the size of this effect (Lee and Penrod, 2022). Within-race differences in appearance could be a meaningful contributor to this variation (Chiroro et al., 2008). Future researchers interested in evaluating how phenotypic bias may affect the ORB should fully cross the design by collecting data from both Black and White participant-witnesses making identifications from both Black and White lineups. Future research should examine whether both Black and White witnesses are similarly affected by phenotypic mismatch.

Finally, facial recognition scholars have spent decades investigating the causes of the own-race bias, but virtually no research has examined why Black suspects are misidentified at higher rates than White suspects. Although scholars suggest that racial disparities in exonerations based on eyewitness misidentifications may be largely explained by an officer’s decision to place Black suspects in lineups when there is little evidence connecting them to the crime (Katzman and Kovera, 2023), lineup construction issues may also contribute. For instance, recent meta-analytic findings suggest that both Black and White witnesses may perform worse on Black than White target lineups (Katzman and Kovera, 2023). Additionally, in a study examining lineup fairness, Black suspects were more likely to be identified from lineups by *both* Black and White mock witnesses (Brigham et al., 1990). Thus, including phenotypic bias as a factor in future investigations could provide (a) greater understanding of the psychological mechanisms responsible for the variations in the size of the ORB, and (b) an explanation for the finding that under certain conditions, Black suspects are at uniquely high risk of being misidentified as the perpetrator of a crime. In the meantime, the findings from this study strongly support that police take care to match the facial phenotype of the suspect when choosing fillers to appear in photo arrays and lineups to eliminate one form of suspect bias.

## Data availability statement

The datasets presented in this study can be found in online repositories. The names of the repository/repositories and accession number(s) can be found below: https://osf.io/am5qh/?view_only=7cf96a56de9946348e001ef2885df2d7.

## Ethics statement

The studies involving humans were approved by John Jay College – City University of New York. The studies were conducted in accordance with the local legislation and institutional requirements. The ethics committee/institutional review board waived the requirement of written informed consent for participation from the participants or the participants’ legal guardians/next of kin because data were collected on the internet so written consent was not possible and would have been the only thing linking participants to their data.

## Author contributions

JMJ conducted most of the data analyses, wrote much of the initial draft of the manuscript, and revised and edited the manuscript. JK provided data analytic support, wrote sections of the initial draft and revision of the manuscript, and edited the manuscript. MBK conceived of the research, secured the funding, oversaw the stimulus creation and data collection, provided the data analytic support, and edited the manuscript. All authors contributed to the article and approved the submitted version.
