# Supplementary material for: Phenotypic mismatch between suspects and fillers but not phenotypic bias increases eyewitness identifications of Black suspects
Source: Front Psychol. 2024 Apr 12;15:1233782. doi: 10.3389/fpsyg.2024.1233782 (PMC11045996; doi:10.3389/fpsyg.2024.1233782)
Supplement: Supplementary file 1 [file Data_Sheet_1.docx]

**Supplemental Materials**

We calculated *d’* and *c* using the following formulas in R (version 4.3.2):

*d*′ = qnorm(hit rate) − qnorm(false alarm rate)

*c* = $-$ $\frac{1}{2}$(qnorm(hit rate) + qnorm(false alarm rate))

The “qnorm” function is part of the base R package and is used to compute z-scores based on the standard normal distribution. The *d’* and *c* scores for each cell of the design and for each of the main effects and interactions are presented in Table S1.

**Table S1**

*Measures of Sensitivity (*d´*) and Bias (*c*) as a Function of Suspect Features, Photo Presentation, and Lineup Composition*

|  | Lineup Composition | | | |  | |
| --- | --- | --- | --- | --- | --- | --- |
|  | Phenotypic Match | | Phenotypic Mismatch | | Collapsed Across Composition | |
|  | *d´* | *c* | *d´* | *c* | *d´* | *c* |
| More Afrocentric | 1.02 | 1.34 | 0.74 | 0.76 | 0.79 | 1.01 |
| Simultaneous | 1.33 | 1.22 | 0.56 | 1.27 | 0.90 | 0.83 |
| Sequential | 0.60 | 1.58 | 0.76 | 0.96 | 0.64 | 1.24 |
| Less Afrocentric | 0.74 | 1.11 | 0.25 | 0.83 | 0.44 | 0.96 |
| Simultaneous | 0.52 | 0.97 | 0.57 | 0.84 | 0.53 | 0.91 |
| Sequential | 1.07 | 1.34 | -0.11 | 0.83 | 0.33 | 1.01 |
| Collapsed across Suspect Features |  |  |  |  |  |  |
| Simultaneous | 0.84 | 1.06 | 0.61 | 0.69 | 0.70 | 0.88 |
| Sequential | 0.80 | 1.50 | 0.30 | 0.89 | 0.46 | 1.11 |
| Collapsed across Presentation | 0.84 | 1.23 | 0.48 | 0.79 | 0.61 | 0.98 |

*Note*. Formulas described above.

**Analysis of Variance on Confidence in Identification Decisions**

To compare means of reported numeric confidence in identification decisions across conditions, we conducted an analysis of variance using IBM’s SPSS Version 28 general linear model univariate procedure. This analysis revealed no significant main effects of photo presentation style, *F*(1, 584) = 2.17, *p* = .142, filler composition, *F*(1, 584) = 0.01, *p* = .916, culprit/suspect phenotype, *F*(1, 584) = 0.77, *p* = .381, nor culprit presence *F*(1, 584) = 0.00, *p* = .984 on participants’ reported numeric confidence in their identification decisions. Mean confidence ratings across all experimental conditions are presented in Table S2. Two-way, three-way, and four-way interactions are reported in Table S3. Only one significant three-way interaction emerged among filler composition, culprit presence, and culprit/suspect phenotype, *F*(1,584) = 7.84, *p* = .005. Pairwise comparisons revealed that in conditions when the fillers matched the culprit/suspect’s phenotype and the guilty culprit was present in the lineup, participant-witnesses were significantly more confident in their identification decisions than when the culprit/suspect presented a less Afrocentric phenotype (*M* = 73.18, *SE* = 2.76) compared to a more Afrocentric phenotype (*M* = 65.38, *SE* = 2.69), *p* = .043.

**Table S2**

*Mean Confidence in Identification Decisions Across Experimental Conditions*

| Photo Presentation | Filler Composition | Culprit/Suspect Phenotype |  | | |  |  | | |
| --- | --- | --- | --- | --- | --- | --- | --- | --- | --- |
|  |  |  | Culprit Absent | | |  | Culprit Present | | |
|  |  |  | *Mean* | *SE* | 95% CI |  | *Mean* | *SE* | 95% CI |
| Simultaneous | Match | Less African | 70.11 | 3.82 | [62.60, 77.61] |  | 78.02 | 3.43 | [71.29, 84.75] |
|  |  | More African | 73.22 | 3.87 | [65.62, 80.83] |  | 63.43 | 3.93 | [55.71, 71.14] |
|  | Mismatch | Less African | 75.13 | 3.77 | [67.73, 82.54] |  | 66.16 | 3.77 | [58.75, 73.56] |
|  |  | More African | 68.36 | 4.17 | [60.16, 76.55] |  | 71.13 | 3.72 | [63.82, 78.44] |
| Sequential | Match | Less African | 63.55 | 3.77 | [56.15, 70.95] |  | 68.35 | 4.32 | [59.87, 76.82] |
|  |  | More African | 69.51 | 3.72 | [62.20, 76.82] |  | 67.33 | 3.68 | [60.11, 74.54] |
|  | Mismatch | Less African | 70.14 | 3.82 | [62.63, 77.64] |  | 69.57 | 3.82 | [62.06, 77.07] |
|  |  | More African | 64.46 | 3.50 | [57.57, 71.34] |  | 70.19 | 3.87 | [62.58, 77.80] |

*Note*. Confidence ratings were given on a 0-100 scale.

**Table S3**

*Tests of Between-Subjects Effects for Two-Way, Three-Way, and Four-Way Interactions on Confidence in Identification Decisions*

| Variables | *F*(1, 584) | *p* |
| --- | --- | --- |
| Lineup Presentation × Filler Composition | 0.40 | .529 |
| Lineup Presentation × Culprit Presence | 1.08 | .299 |
| Lineup Presentation × Suspect Phenotype | 0.74 | .389 |
| Filler Composition × Culprit Presence | 0.01 | .909 |
| Filler Composition × Suspect Phenotype | 0.00 | .983 |
| Culprit Presence × Suspect Phenotype | 0.19 | .664 |
| Lineup Presentation × Filler Composition × Culprit Presence | 0.20 | .652 |
| Lineup Presentation × Filler Composition × Suspect Phenotype | 1.66 | .198 |
| Lineup Presentation × Culprit Presence × Suspect Phenotype | 0.12 | .729 |
| Filler Composition × Culprit Presence × Suspect Phenotype | 7.84 | .005 |
| Lineup Presentation × Filler Composition × Culprit Presence × Suspect Phenotype | 1.12 | .290 |

**Analysis of Variance on Confidence in Suspect Identifications**

Next, we compared mean confidence ratings for suspect identifications specifically by selecting only cases where participants made an identification of our designated suspect. Mean confidence ratings across all experimental conditions are presented in Table S4. Again, this analysis revealed no significant main effects of photo presentation style, *F*(1, 88) = 2.73, *p* = .113, filler composition, *F*(1, 88) = 0.02, *p* = .856, culprit/suspect phenotype, *F*(1, 88) = 0.49, *p* = .456, nor culprit presence, *F*(1, 88) = 0.20, *p* = .696 on participants’ reported numeric confidence in their suspect identification decisions. Two-way, three-way, and four-way interactions are reported in Table S5. The same significant three-way interaction emerged between filler composition, culprit presence, and culprit/suspect phenotype, *F*(1, 88) = 5.11, *p* = .023. Pairwise comparisons revealed that in conditions when the guilty culprit was present in the lineup and fillers matched the culprit/suspect’s phenotype, participants made more confident suspect identifications when the culprit/suspect presented a less Afrocentric phenotype (*M* = 76.74, *SE* = 5.55) compared to a more Afrocentric phenotype (*M* = 59.25, *SE* = 6.47), *p* = .043]. In conditions when the fillers mismatched the culprit/suspect’s phenotype and the culprit/suspect presented a more Afrocentric phenotype, participants’ confidence in their suspect identifications was significantly higher when the guilty culprit was present (*M* = 79.29, *SE* = 4.41), compared to when designated suspect was innocent (*M* = 56.75, *SE* = *7*.06), *p* = .008. Finally, in conditions when the guilty culprit was present in the lineup and the culprit/suspect presented a more Afrocentric phenotype, participants made more confident suspect identifications when the fillers were mismatched to the culprit/suspect’s phenotype (*M* = 79.29, *SE* = 4.41) than when fillers were matched to the suspect’s phenotype (*M* = 59.25 *SE* = 6.47), *p* = .009.

**Table S4**

*Mean Confidence in Suspect Identifications Across Experimental Conditions*

| Photo Presentation | Filler Composition | Culprit/Suspect Phenotype |  | | |  |  | | |
| --- | --- | --- | --- | --- | --- | --- | --- | --- | --- |
|  |  |  | Culprit Absent | | |  | Culprit Present | | |
|  |  |  | *Mean* | *SE* | 95% CI |  | *Mean* | *SE* | 95% CI |
| Simultaneous | Match | Less African | 81.50 | 10.94 | [59.76,103.24] |  | 80.64 | 6.60 | [67.53, 93.74] |
|  |  | More African | 78.00 | 21.88 | [34.53, 121.47] |  | 64.50 | 6.92 | [50.75, 78.25] |
|  | Mismatch | Less African | 73.80 | 9.78 | [54.36, 93.24] |  | 72.82 | 6.60 | [59.71, 85.93] |
|  |  | More African | 67.50 | 8.93 | [49.75, 85.25] |  | 77.38 | 5.47 | [66.51, 88.24] |
| Sequential | Match | Less African | 51.00 | 21.88 | [7.53, 94.47] |  | 72.83 | 8.93 | [55.09, 90.58] |
|  |  | More African | 70.00 | 21.88 | [26.53, 113.47] |  | 54.00 | 10.94 | [32.26, 75.74] |
|  | Mismatch | Less African | 77.13 | 7.74 | [61.76, 92.50] |  | 63.14 | 8.27 | [46.71, 79.57] |
|  |  | More African | 46.00 | 10.94 | [24.26, 67.74] |  | 81.20 | 6.92 | [67.45, 94.95] |

*Note*. Confidence ratings were given on a 0-100 scale.

**Table S5**

*Tests of Between-Subjects Effects for Two-Way, Three-Way, and Four-Way Interactions on Confidence in Suspect Identifications*

| Variables | *F* | *p* |
| --- | --- | --- |
| Lineup Presentation × Filler Composition | 0.45 | .536 |
| Lineup Presentation × Culprit Presence | 0.44 | .477 |
| Lineup Presentation × Suspect Phenotype | 0.03 | .825 |
| Filler Composition × Culprit Presence | 0.62 | .404 |
| Filler Composition × Suspect Phenotype | 0.01 | .886 |
| Culprit Presence × Suspect Phenotype | 0.04 | .884 |
| Lineup Presentation × Filler Composition × Culprit Presence | 0.03 | .835 |
| Lineup Presentation × Filler Composition × Suspect Phenotype | 0.41 | .495 |
| Lineup Presentation × Culprit Presence × Suspect Phenotype | 0.07 | .752 |
| Filler Composition × Culprit Presence × Suspect Phenotype | 5.11 | .026 |
| Lineup Presentation × Filler Composition × Culprit Presence × Suspect Phenotype | 1.69 | .215 |

**Confidence Accuracy Characteristic (CAC) Analyses**

To determine the accuracy of high-confident suspect identifications in the phenotypic match vs. phenotypic mismatch conditions, we plotted confidence-accuracy characteristic (CAC) curves (Mickes, 2015). In a CAC curve, the accuracy of suspect identifications is calculated for each confidence bin, and accuracy at each confidence level is computed as follows:

Accuracy $=\frac{suspect IDs in culprit present lineups}{suspect IDS in culprit present lineups + suspect IDS in culprit absent lineups}$

Because of the small number of responses in some bins, and because we were primarily interested in the reliability of high confident suspect identifications, low confidence responses were collapsed into a 0-59 bin, as has been done in prior research (Mickes, 2015). Although no formal method exists for determining sample size in CAC analysis, prior researchers have aimed for at least 40 observations in the high-confidence bins for each condition (Giacona et al., 2021). Because our *N* is far below this value, our estimates are unstable for this exploratory analysis. However, the pattern of results in Figure S1 provides additional evidence that phenotypic mismatched lineups are problematic for the legal system. Specifically, these data demonstrate that highly confident suspect identifications can be produced via suspect bias rather than a recognition memory process. More witnesses make suspect identifications from mismatched lineups than matched lineups at every confidence level, and this is particularly true for those in the high confidence (90-100) bin.

| **Figure S1**  *Confidence Accuracy Characteristic Curves for the Phenotypic Match and Phenotypic Mismatch Conditions*  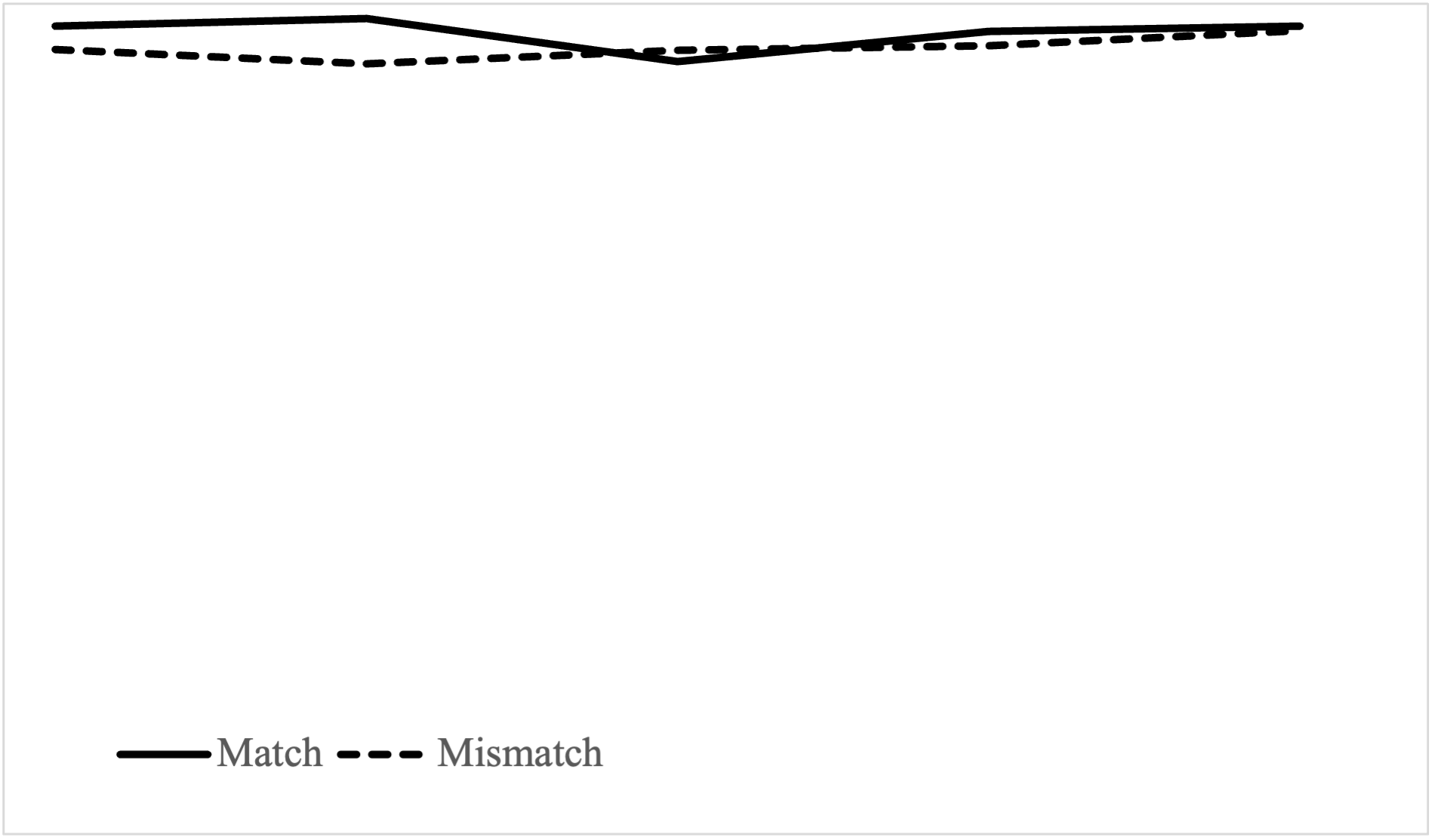 | |
| --- | --- |
| 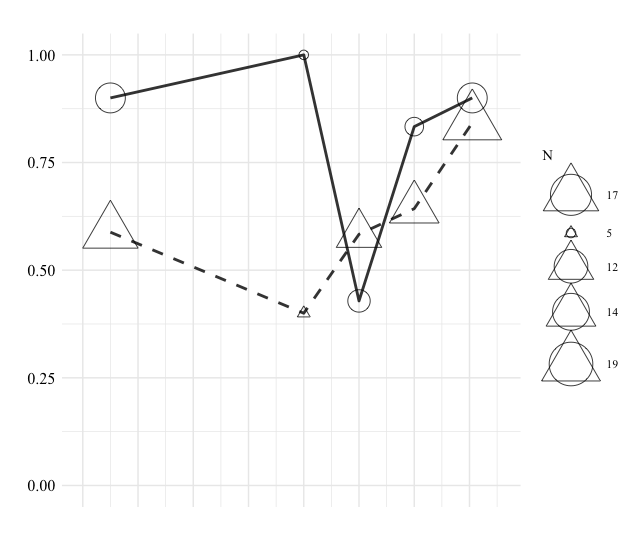  **Accuracy** | |
|  | 0-59 60-69 70-79 80-89 90-100 |
| **Confidence (%)** | |

**Higher Order Effects for Regression Analyses on Identification Decisions**

**Table S6**

*Regression Analysis Summary: Suspect Identifications*

| Predictors | *B* | *SE* | *Wald’s X^2^*  (1, *N* = 600) | 95% CI  [LL, UL] | *p* | *OR* |
| --- | --- | --- | --- | --- | --- | --- |
| Suspect/Culprit Phenotype | -0.73 | 0.62 | 1.42 | [0.15, 1.60] | .233 | 0.48 |
| Filler Composition | 0.88 | 0.56 | 2.49 | [0.81, 7.24] | .115 | 2.42 |
| Lineup Presentation | -0.36 | 0.62 | 0.35 | [0.21, 2.32] | .557 | 0.70 |
| Culprit Presence | 1.45 | 0.51 | 8.02 | [1.56, 11.61] | .005 | 4.26 |
| Filler Composition × Lineup Presentation | 0.43 | 0.65 | 0.43 | [0.43, 5.49] | .510 | 1.53 |
| Culprit Presence × Lineup Presentation | -0.21 | 0.50 | 0.17 | [0.31, 2.15] | .678 | 0.81 |
| Suspect/Culprit Phenotype × Lineup Presentation | -0.56 | 0.78 | 0.51 | [0.13, 2.62] | .474 | 0.57 |
| Suspect/Culprit Phenotype × Filler Composition | 0.74 | 0.61 | 1.50 | [0.64, 6.93] | .221 | 2.11 |
| Suspect/Culprit Phenotype × Culprit Presence | 0.78 | 0.50 | 2.45 | [.82, 5.82] | .118 | 2.19 |
| Culprit Presence × Filler Composition | -0.94 | 0.54 | 3.03 | [0.14, 1.13] | .082 | 0.39 |
| Suspect Phenotype × Filler Composition × Lineup Presentation | -0.07 | 0.96 | 0.01 | [0.14, 6.16] | .946 | 0.94 |

*Note*. The table presents the regression results from the hierarchical binary logistic regression after the third block was entered. All four main effects were entered in the first block, followed by all six two-way interactions in the second block, and our predicted three-way interaction in the third block. Adding interaction terms did not improve model fit. Statistics for the main effects from the first block of the regression (main effects only) are provided in the manuscript.

**Table S7**

*Regression Analysis Summary: Filler Identifications*

| Predictors | *B* | *SE* | *Wald’s X^2^*  (1, *N* = 600) | 95% CI  [LL, UL] | *p* | *OR* |
| --- | --- | --- | --- | --- | --- | --- |
| Suspect/Culprit Phenotype | -0.10 | 0.38 | 0.07 | [0.43, 1.90] | .799 | 0.91 |
| Filler Composition | -0.41 | 0.38 | 1.11 | [0.31, 1.42] | .291 | 0.67 |
| Lineup Presentation | 0.25 | 0.38 | 0.43 | [0.61, 2.66] | .514 | 1.28 |
| Culprit Presence | -0.26 | 0.33 | 0.63 | [0.40, 1.48] | .429 | 0.77 |
| Filler Composition × Lineup Presentation | -0.18 | 0.49 | 0.13 | [0.32, 2.20] | .716 | 0.84 |
| Culprit Presence × Lineup Presentation | 0.22 | 0.35 | 0.39 | [0.63, 2.44] | .534 | 1.24 |
| Suspect/Culprit Phenotype × Lineup Presentation | 0.31 | 0.47 | 0.42 | [0.54, 3.42] | .519 | 1.36 |
| Suspect/Culprit Phenotype × Filler Composition | -0.16 | 0.50 | 0.11 | [0.32, 2.28] | .744 | 0.85 |
| Suspect/Culprit Phenotype × Culprit Presence | 0.35 | 0.35 | 1.00 | [0.72, 2.78] | .317 | 1.41 |
| Culprit Presence × Filler Composition | -0.12 | 0.35 | 0.12 | [0.45, 1.74] | .725 | 0.89 |
| Suspect Phenotype × Filler Composition × Lineup Presentation | 0.18 | 0.69 | 0.07 | [0.31, 4.67] | .791 | 1.20 |

*Note*. The table presents the regression results from the hierarchical binary logistic regression after the third block was entered. All four main effects were entered in the first block, followed by all six two-way interactions in the second block, and our predicted three-way interaction in the third block. Adding interaction terms did not improve model fit. Statistics for the main effects from the first block of the regression (main effects only) are provided in the manuscript.

**Table S8**

*Regression Analysis Summary: Lineup Rejections*

| Predictors | *B* | *SE* | *Wald’s X^2^*  (1, *N* = 600) | 95% CI  [LL, UL] | *p* | *OR* |
| --- | --- | --- | --- | --- | --- | --- |
| Suspect/Culprit Phenotype | 0.31 | 0.37 | 0.67 | [0.65, 2.82] | .412 | 1.36 |
| Filler Composition | 0.15 | 0.37 | 0.17 | [0.57, 2.38] | .678 | 1.16 |
| Lineup Presentation | -0.17 | 0.37 | 0.21 | [0.41, 1.75] | .647 | 0.84 |
| Culprit Presence | -0.42 | 0.33 | 1.65 | [0.35, 1.25] | .198 | 0.66 |
| Filler Composition × Lineup Presentation | -0.02 | 0.47 | 0.00 | [0.39, 2.46] | .975 | 0.99 |
| Culprit Presence × Lineup Presentation | 0.16 | 0.34 | 0.21 | [0.60, 2.26] | .644 | 1.17 |
| Suspect/Culprit Phenotype × Lineup Presentation | -0.20 | 0.48 | 0.17 | [0.32, 2.10] | .679 | 0.82 |
| Suspect/Culprit Phenotype × Filler Composition | -0.28 | 0.47 | 0.35 | [0.30, 1.92] | .556 | 0.76 |
| Suspect/Culprit Phenotype × Culprit Presence | -0.66 | 0.34 | 3.84 | [0.27, 1.00] | .050 | 0.52 |
| Culprit Presence × Filler Composition | 0.20 | 0.34 | 0.34 | [0.63, 2.35] | .557 | 1.22 |
| Suspect Phenotype × Filler Composition × Lineup Presentation | 0.21 | 0.67 | 0.09 | [0.33, 4.57] | .759 | 1.23 |

*Note*. Lineup rejections include both “Not Present” and “Don’t Know” responses. The table presents the regression results from the hierarchical binary logistic regression after the third block was entered. All four main effects were entered in the first block, followed by all six two-way interactions in the second block, and our predicted three-way interaction in the third block. Adding interaction terms did not improve model fit. Statistics for the main effects from the first block of the regression (main effects only) are provided in the manuscript.

**References**

Giacona, A. M., Lampinen, J. M., & Anastasi, J. S. (2021). Estimator variables can matter even for high-confidence lineup identifications made under pristine conditions. Law and Human Behavior, 45(3), 256–270. https://doi.org/10.1037/lhb0000381

Mickes, L. (2015). Receiver operating characteristic analysis and confidence–accuracy characteristic analysis in investigations of system variables and estimator variables that affect eyewitness memory. Journal of Applied Research in Memory and Cognition, 4(2), 93–102. https://doi.org/10.1016/j.jarmac.2015.01.003
